# Effectiveness of skills-based training using the Drink-less package to increase family practitioner confidence in intervening for alcohol use disorders

**DOI:** 10.1186/1472-6920-6-8

**Published:** 2006-02-06

**Authors:** Elizabeth M Proude, Katherine M Conigrave, Paul S Haber

**Affiliations:** 1 Drug Health Services, Royal Prince Alfred Hospital, Camperdown, New South Wales, Australia; 2 School of Public Health, University of Sydney, Sydney, New South Wales, Australia; 3 Discipline of Medicine, University of Sydney, Sydney, New South Wales, Australia; 4 Discipline of Psychological Medicine, University of Sydney, Sydney, New South Wales, Australia

## Abstract

**Background:**

Misuse of alcohol is second only to tobacco as a leading cause of preventable death in Australia. There is an opportunity in family practice to detect problems and intervene with people at risk of alcohol-related harm before complications occur. However, family practitioners (FPs) report low levels of confidence in managing patients with drinking problems. The aim of this study was to determine whether the interactive training session using the 'Drink-less' package led to improvement in FPs' self-reported level of confidence in detecting and providing interventions for risky alcohol consumption.

**Method:**

FPs in urban and rural New South Wales were invited to training sessions in their local area. An introductory overview preceded a practical skills- based session, using the Drink-less package. Participants completed before and after evaluation forms.

**Results:**

While 49% (CI 43 – 55) of the attending FPs indicated at baseline that they felt confident in identifying at-risk drinkers, this proportion rose to 90% (95% CI: 87 – 93) post-session, and they also reported increases in confidence from 36% (95% CI: 31 – 41) to 90% in their ability to advise patients. Urban FPs reported lower levels of confidence than rural FPs, both pre- and post-session.

**Conclusion:**

Training sessions in the Drink-less intervention resulted in increased self-reported confidence in detection and brief intervention for alcohol problems. Further research is needed to determine the duration of this effect and its influence on practice behaviour.

## Background

The misuse of alcohol is second only to tobacco as a leading cause of preventable death in Australia, [1] and one in ten adults are at long-term risk of harm from their alcohol consumption [2-4]. There is good evidence that brief interventions are effective in reducing alcohol consumption and related problems, particularly in non-dependent drinkers, [5-8] and are a cost-effective technique [9]. However, many physicians do not routinely screen or advise patients [10].

The first point of contact with the health system for a patient is typically the family practitioner (FP). Eighty-five per cent of the Australian population attend a family practice at least annually and people who seek help for a drinking problem are most likely to talk to an FP first [11,12]. Therefore the opportunity exists to detect and intervene with people at risk of alcohol-related harm before complications occur and drinking patterns become entrenched.

However, FPs remain reluctant to undertake systematic screening and intervention for risky alcohol consumption [13]. Detection rates for alcohol problems have remained low, [14,15] despite nearly 20 years of evidence of effectiveness of early intervention and attempts at changing medical education. In recent surveys, FPs still detect or offer advice on as few as 23% of alcohol problems, similar to levels detected in the 1980's [10,16].

FPs report that time constraints, lack of confidence, fear of intrusiveness, skepticism about achieving results, and sometimes financial disincentives, are major barriers to improved detection [13,17,18]. They are asked to undertake preventive medicine amidst increasing workloads and despite the conventional view of the consultation being for diagnosis and treatment of presenting problems. FPs, especially in rural areas, are already under considerable pressure due to insufficient numbers and unfavourable cost structures. More can be done to enhance the delivery of the most effective detection and intervention techniques [19].

The World Health Organization has been involved in a series of studies aimed at facilitating detection and brief intervention for hazardous drinking. In the second phase of this work, the WHO multi-centre trial demonstrated that as little as five minutes of advice was associated with significant reductions in drinking at 6 months follow-up [5]. The screening and brief intervention tools were subsequently adapted for use in the routine clinical setting. In Australia these tools were packaged together in 1993 into a user-friendly kit for FPs, known as 'Drink-less' by Gomel, Saunders et al [20]. It originally included an Australian modification of the Alcohol Use Disorders Identification Test (known as AUDIT) as well as intervention materials based on the five-minute intervention technique in the WHO collaborative trial [21,5].

The Drink-less package provides a laminated card to guide the FP through the intervention. It describes the prevalence of excessive drinking, likely harms from drinking, likely benefits from reduced drinking and suggested approaches to control drinking. The package has been used in Australian family practice since its development. Focus group feedback from FPs who had used the package indicated it made management of alcohol problems less daunting. The Drink-less materials were updated in 2003 to include the original AUDIT questionnaire rather than the modified screening tool, and so that drinking goals suggested in the intervention better matched revised Australian guidelines. The layout was also redesigned and updated [22].

In this study we determine whether an interactive skills-based training session using the Drink-less package led to measurable changes in FPs' self-reported level of confidence in detecting and providing interventions for risky alcohol consumption.

## Method

This project was conducted in the context of a program from the Roads & Traffic Authority of New South Wales (RTA) to train FPs in brief intervention in conjunction with a new Alcohol Ignition Interlock Program for drink drivers. Family practitioners throughout urban and rural New South Wales were invited to evening training sessions though their local Divisions of General Practice. The Divisions of General Practice were responsible for organising educational events for their members according to demand, interest and the availability of presenters, and sending out invitations to member lists that are not available to outside individuals or organisations. To increase the appeal of training sessions, we applied to The Royal Australian College of General Practitioners and to the Australian College of Rural & Remote Medicine for continuing education (CME) points for participants. In addition, training activities were conducted after a complimentary restaurant dinner, with a guest expert speaker. In rural areas, other guests such as practice nurses, emergency department staff, ambulance officers and pharmacists were also invited as part of a community project that was taking place in some of the smaller towns in 2005.

The program consisted of an introductory one-hour session 'Alcohol use disorders: update on assessment and management', which included an overview on detection and diagnosis of alcohol use disorders, from hazardous use through to dependence, outpatient management of alcohol withdrawal, and new pharmacotherapies for relapse prevention. This was largely a didactic session. After a brief description of the Alcohol Interlock program commencing in the State, the next 45 minutes was an interactive skills-based training session centred on the use of the Drink-less package. Participants were trained in scoring the AUDIT, in advising the patient on drinking, arranging for ongoing treatment including pharmacotherapy for dependent cases, indications for referral, and planning follow-up. Interactive discussions of case studies illustrated the use of the package. Further informal discussions took place after the activity. The presentations were led by a local drug and alcohol specialist, by one of the authors (KC or PH) or by another Fellow from the Chapter of Addiction Medicine, Royal Australian College of Physicians.

Pre and post- surveys were distributed to all participants with matched questions appearing in both surveys (Table 1). In the post-survey, an extra question asked 'how confident do you feel about your understanding of the requirements of the brief medical intervention for the RTA Interlock program?' Response categories were the same as before for all questions. No demographic information was collected on the surveys in order to reduce social desirability bias and protect confidentiality; in a rural area with only three or four doctors it would have been easy to identify the respondents.

Data from these surveys were entered and analysed in SPSS v12. Chi square analyses were carried out to compare the responses between rural and urban FPs. As the data were not normally distributed, Wilcoxon signed ranks tests were used to compare pre- and post- training survey responses. Statistical significance was determined as p < 0.05 at a 95% confidence interval. Responses to open questions were recorded, subjected to content analysis and grouped into emerging themes.

FPs who were willing to accept referrals from the Roads & Traffic Authority for the Interlock Program brief medical intervention completed separate consent forms.

## Results

Twenty-four training sessions were conducted over 2003, 2004 and 2005 with a total of 424 people attending, of whom 419 completed evaluation forms. Responders were 300 (73%) FPs, with 112 in other categories, including medical student, nurse, pharmacist, ambulance officer, psychologist or counsellor, drug and alcohol worker, and probation and parole officer. Not all respondents answered all questions. Analysis of the results below is confined to FPs (n = 300). One hundred and sixty-nine (56%) FPs were from urban areas and 131 (44%) from rural areas.

The learning expectations the doctors described covered three general areas: information; identification and assessment skills; and intervention and management skills. A selection of typical responses is grouped under these headings. Among 'information needs', responses included 'what drinking levels are safe?' 'what is problem drinking?' 'how to manage problem drinking?' Needs expressed for identification and treatment included 'latest methods of assessment and intervention', 'how do I get the message through?' 'techniques to identify and approach a problem drinker', 'what to do with drinkers who deny their dependence?' Under 'intervention and management skills', FPs expressed they wanted strategies to use on recalcitrant or relapsing patients, a quick and effective way of intervening, strategies to use with relapse and problems such as family difficulties, work problems, and drink driving; and management of hazardous intake, dependence, and withdrawal.

Other expectations voiced were 'obtaining information about drinking and driving, with emphasis on the RTA's new initiative'; update on the new medications for treatment of alcohol problems; indications for detoxification, and the detox sedation regimen; other drug withdrawal management, and information about local followup and support services. Networking with colleagues was also mentioned.

Of the 199 separate consent forms collected from doctors, 142 (71%) agreed to being on the RTA list as a provider of brief intervention to drink-drive offenders entering the Interlock Program. Thirteen were not eligible (due to reasons such as imminent retirement, maternity leave, registrar or locum status) and 44 declined.

### Post-survey

**Figure 1:**
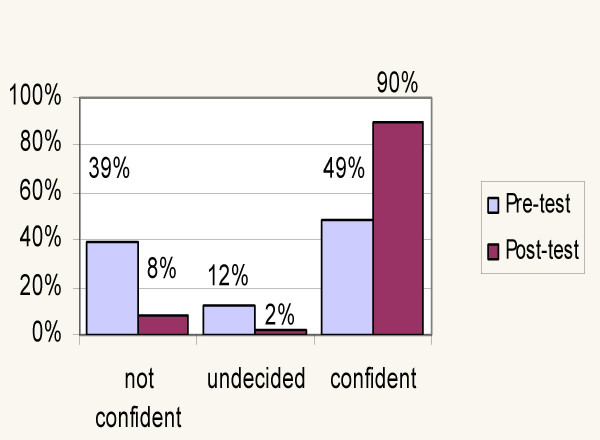
Ability to identify at-risk drinkers.

**Figure 2:**
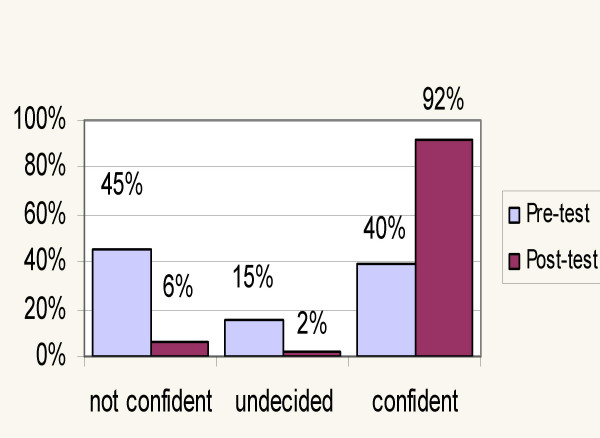
Deciding the steps to take with at-risk drinkers.

**Figure 3:**
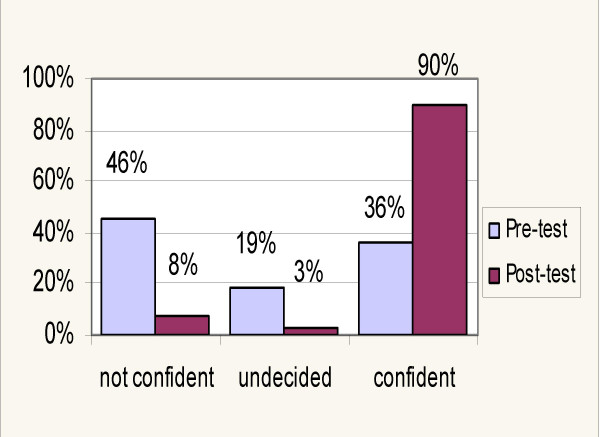
Conducting brief intervention for risky alcohol use.

At pre-test, 7% (95% CI: 4 to 10) of FPs felt 'not at all confident' at identifying problem drinkers, 11% (95% CI: 7 to 14) were 'not at all confident' in deciding what steps to take next, and 13% (95% CI: 9 to 17) were not at all confident at carrying out a brief intervention, whereas at post-test no-one responded this way. In further analyses the five response categories were collapsed into 'not confident', 'undecided' and 'confident' (Figures 1, 2, 3). While 49% (95% CI: 43 to 55) of the FPs attending our training sessions indicated at baseline that they felt confident in identifying at-risk drinkers, this proportion rose to 90% (95% CI: 87 to 93) post-session. They also reported increased confidence in their ability to advise patients, which rose from 36% (95% CI: 31 to 41) to 90% (95% CI: 87 to 93). These differences were all statistically significant at p < 0.000. On comparing responses between urban and rural FPs, the rural FPs showed significantly higher levels of confidence than the urban FPs both at pre- and post-test over all items (p = 0.001), except for their confidence in ability to conduct brief intervention at pre-test (p = 0.123). The rural FPs' level of confidence rose from 42% to 97% on this item while the urban FPs rose from 31% to 84%. However, in both rural and urban FPs the rise in confidence remained significant and did not differ between the groups.

Positive comments about the workshop included comments such as 'good combination of theory and practice; very relevant to family practice; case studies helpful; excellent presentation of a rarely discussed problem'. Other statements mentioned the practical nature of the 'helpful, easy to use materials' and the importance of identifying not only high-risk but also low-risk drinkers 'as prevention is extremely important'. Negative comments and suggestions included 'how to initiate alcohol reduction apart from losing licence?; not sure how negative patients will be with the Interlock program; would like a full workshop on the intervention; still a difficult issue to effectively address'.

## Discussion

This skills-based training program using WHO detection and intervention materials, packaged in the user-friendly 'Drink-less' kit, resulted in significant increases in self-report of FP confidence in detection and management of alcohol problems. While 49% of the FPs attending our training sessions indicated at baseline that they felt confident in identifying at-risk drinkers, this proportion rose to 90% post-session, and confidence also increased in ability to advise risky and high-risk drinkers.

It has been found that even structured advice of five minutes' duration results in a statistically significant and clinically relevant improvement in drinking [3,6,8,23]. However, while FPs are willing to treat alcohol problems, [16] they are reported as sometimes reluctant to initiate discussion of non-presenting problems, especially in the difficult alcohol and drug area [24]. They may consider such patients unmotivated to change, and the success rate low [25]. Further, a survey of FPs in Sydney found that more than a quarter were unaware of the safe drinking levels for men or women or the appropriate treatment for patients consuming above such levels [16].

The Drink-less package provides a quick way to screen patients and provide a structured and quick intervention. The Roads & Traffic Authority of New South Wales obliges all drivers who have been given the opportunity of the Alcohol Interlock Program as part of a reduced penalty for a drink driving offence to have a brief medical intervention, standardised by the use of the Drink-less package. Hence, the RTA needs FPs in all areas of the State to be trained in this brief intervention technique. This link between health and law enforcement provided the opportunity to roll out State-wide training for FPs, and these FPs have so far been referred several hundred patients. This provides the chance to practice brief intervention skills, regardless of whether they have already adopted this as routine clinical practice. It is also hoped that increased confidence resulting from training would encourage FPs to undertake systematic screening and intervention with all patients. Other studies report that while more experience results in increases in confidence, [26] more training does not always produce the same effect [27].

The main limitations of the study are that the sample comprised self-selected and motivated attendees and data is not available on numbers of FPs who were invited but did not attend. Nonetheless, attendees reported low levels of confidence in this area at the start of the training activity, especially among urban FPs. This was a before and after study on the same group of people who served as their own controls, with no comparison group who did not receive training. We are only able to describe the participants' self-reported confidence in identifying and providing interventions with heavy drinkers and we have no evidence that the changes in confidence were carried through into practice. It is possible also that attrition of the effect of teaching on confidence may occur over time, especially if the screening procedure (and thus the intervention) is not adopted into routine practice. The current session was a skills-based one and so provided experience in a theoretical scenario. It is likely that reinforcement of learned skills in follow-up sessions will be required.

Demand for these training sessions remains constant, especially in country areas. The traditional medical curriculum included relatively small content on diagnosis and treatment of alcohol problems, especially the early stages, and negative attitudes to alcohol problems were possibly generated by the exposure of medical students almost exclusively to hospitalised patients with late complications of dependence [28]. Practical skills in providing advice on alcohol were typically not part of the curriculum. Even now, coverage of this area tends to vary between universities in Australia.

In other countries, practice nurses take on screening and brief intervention and this would be especially useful in rural and remote areas where there are shortages of doctors. In addition, time saving techniques such as waiting room screening including using handheld computers are being investigated.

## Conclusion

Skills-based training that includes use of a validated screening tool such as AUDIT, in conjunction with user-friendly and validated aids to brief intervention and other treatment, can increase FPs' confidence in detecting and treating alcohol problems. There is a need for more research into methods of screening and the effect of experience and followup booster sessions on actual implementation of screening and intervention in routine practice.

## Competing interests

The author(s) declare that they have no competing interests.

## Authors' contributions

All authors contributed to the design of this study, the design and implementation of the training sessions and took part as presenters in the training sessions. EP entered and analysed the data and prepared the first draft of the manuscript; all authors contributed equally to the writing of the manuscript and read and approved the final version.

## Pre-publication history

The pre-publication history for this paper can be accessed here:



## Figures and Tables

**Table 1:** Survey questions

**Pre-session**	**Response categories**
*How confident do you feel about*: • Your ability to identify at-risk drinkers? • Your ability to decide which steps to take after identifying at-risk drinkers? • Your ability to conduct brief interventions for alcohol problems?	Not at all confident, somewhat confident, undecided, fairly confident, very confident
What do you expect to learn from today's workshop?	(open response)
**Post-session**	
• *How confident do you feel about:* • Your ability to identify at-risk drinkers? • Your ability to interpret AUDIT scores? • Your ability to decide which steps to take after identifying at-risk drinkers? • Your ability to conduct a brief intervention for alcohol problems? • Your understanding of the requirements of the brief medical intervention for the RTA Alcohol Interlock Program?	Not at all confident, somewhat confident, undecided, fairly confident, very confident
Any comments about the workshop?	(open response)

## References

[B1] Australia C (2001). National Alcohol Strategy: a plan for action 2001 to 2004.

[B2] AIHW (2005). 2004 National Drug Strategy Household Survey. First results. AIHW cat no. PHE 57.

[B3] Wilk AI, Jensen NM, Havighurst TC (1997). Meta-analysis of randomized control trials addressing brief interventions in heavy alcohol  drinkers.. Journal of General Internal Medicine.

[B4] Teesson M, Hall W, Lynskey M, Degenhardt L (2000). Alcohol- and drug-use disorders in Australia: implications of the National Survey of Mental Health and WellBeing. Aust N Z J Psychiatry.

[B5] WHO Brief Intervention Study Group (1996). A cross-national trial of brief interventions with heavy drinkers. American Journal of Public Health.

[B6] Bien TH, Miller WR, Tonigan JS (1993). Brief interventions for alcohol problems: a review. Addiction.

[B7] Wutzke SE, Conigrave KM, Saunders JB, Hall WD (2002). The long term effectiveness of brief interventions for unsafe alcohol consumption: a 10 year follow-up. Addiction.

[B8] Moyer A, Vergun P, Finney JW, Swearingen CE (2002). Brief interventions for alcohol problems: a meta-analytic review of controlled investigations in treatment-seeking and non-treatment-seeking populations.. Addiction.

[B9] Wutzke SE, Shiell A, Gomel MK, Conigrave KM (2001). Brief interventions for alcohol: low outlays, high exposure and demonstrable effects.  But are they cost-effective?. Social Science and Medicine.

[B10] Denny CH, Nelson DE, Serdula MK, Holtzman D (2003). Physician advice about smoking and drinking: are U.S. adults being informed?. American Journal of Preventive Medicine.

[B11] Valenti L, Harrison C, Britt H, Miller GC, Knox S, Charles J, Henderson J, Pan Y, Bayram C (2003). General practice activity in Australia 2002-03. AIHW Cat. No. GEP 16..

[B12] Proudfoot H, Teesson M (2002). Who seeks treatment for alcohol dependence? Findings from the Australian National Survey of Mental Health and Wellbeing. Social Psychiatry & Psychiatric Epidemiology.

[B13] Beich A, Malterud K, Gannick D (2002). Screening and brief intervention for excessive alcohol use: qualitative interview study of the experiences of general practitioners.. British Medical Journal.

[B14] Reid ALA, Webb GR, Hennrikus D, Fahey PP, Sanson-Fisher RW (1986). General practitioners' detection of patients with high alcohol intake. British Medical Journal.

[B15] Aira M, Kauhanen J, Larivaara P, Rautio P (2003). Factors influencing inquiry about patients' alcohol consumption by primary health care physicians: qualitative semi-structured interview study.. Fam Pract.

[B16] Fucito LM, Haber PS, Gomes BS, Murnion B (2003). General practitioners' diagnostic skills and referral practices in managing patients with drug and alcohol-related health problems: implications for medical training and education programmes.. Drug & Alcohol Review.

[B17] Spandorfer JM, Turner BJ, Israel Y (1999). Primary care physicians' views on screening and management of alcohol abuse: inconsistencies with national guidelines.. Journal of Family Practice.

[B18] Brotons C, Lionis C, Bjorkelund C, Bulc M, Ciurana R, Godycki-Cwirko M, Jurgova E, Kloppe P (2005). Prevention and health promotion in clinical practice: the views of general practitioners in Europe.. Preventive Medicine.

[B19] Whitlock EP, Allan J, Orleans T, Pender N (2002). Evaluating primary care behavioral counseling interventions. An evidence-based approach.. American Journal of Preventive Medicine.

[B20] Gomel MK, Saunders JB, Burns L, Hardcastle DM, Sumich M (1994). Dissemination of early intervention for harmful alcohol consumption in general practice. Health Promotion Journal of Australia.

[B21] Saunders JB, Grant M, Aasland OG, Babor TF, de la Fuente Juan R (1993). Development of the Alcohol Use Disorders Identification Test (AUDIT): WHO Collaborative Project on Early Detection of Persons with Harmful Alcohol Consumption - II. Addiction.

[B22] The Drink-less Program.  http://www.cs.nsw.gov.au/drugahol/drinkless/. http://www.cs.nsw.gov.au/drugahol/drinkless/.

[B23] Fleming MF, London R, Barry KL, Manwell LB, Johnson K (1997). Brief physician advice for problem alcohol drinkers: a randomized controlled trial in community-based primary care practices. JAMA.

[B24] Miller NS, Sheppard LM, Colenda CC, Magen J (2001). Why physicians are unprepared to treat patients who have alcohol- and drug-related disorders. Academic Medicine.

[B25] Richmond RL, Roche AM, Hotham ED (2002). The general practitioner's role on AOD issues: overcoming individual, professional and systemic barriers.. Drug & Alcohol Review.

[B26] Kaner EF, Bouix JC, Wutzke S, Saunders JB, Powell A, Morawski J (2001). Impact of alcohol education and training on general practitioners' diagnostic and management skills: findings from a World Health Organization collaborative study. Journal of Studies on Alcohol.

[B27] Babor TF, Higgins-Biddle JC, Gould BE, Higgins PS, Gassman RA (2004). Training medical providers to conduct alcohol screening and brief interventions.. Substance Abuse.

[B28] Wallace P (2000). Medical students, drugs and alcohol: time for medical schools to take the issue seriously.. Medical Education.

